# Genome-Wide Pleiotropy of Osteoporosis-Related Phenotypes: The Framingham Study

**DOI:** 10.1002/jbmr.38

**Published:** 2010-01-29

**Authors:** David Karasik, Yi-Hsiang Hsu, Yanhua Zhou, L Adrienne Cupples, Douglas P Kiel, Serkalem Demissie

**Affiliations:** 1 Hebrew SeniorLife Institute for Aging Research and Harvard Medical SchoolBoston, MA, USA; 2 Department of Biostatistics, Boston University School of Public HealthBoston, MA, USA

**Keywords:** bone mineral density, quantitative ultrasound, femoral geometry, genome-wide association, single-nucleotide polymorphisms, genetic correlations, pleiotropy

## Abstract

Genome-wide association studies offer an unbiased approach to identify new candidate genes for osteoporosis. We examined the Affymetrix 500K + 50K SNP GeneChip marker sets for associations with multiple osteoporosis-related traits at various skeletal sites, including bone mineral density (BMD, hip and spine), heel ultrasound, and hip geometric indices in the Framingham Osteoporosis Study. We evaluated 433,510 single-nucleotide polymorphisms (SNPs) in 2073 women (mean age 65 years), members of two-generational families. Variance components analysis was performed to estimate phenotypic, genetic, and environmental correlations (ρ_P_, ρ_G_, and ρ_E_) among bone traits. Linear mixed-effects models were used to test associations between SNPs and multivariable-adjusted trait values. We evaluated the proportion of SNPs associated with pairs of the traits at a nominal significance threshold α = 0.01. We found substantial correlation between the proportion of associated SNPs and the ρ_P_ and ρ_G_ (*r* = 0.91 and 0.84, respectively) but much lower with ρ_E_ (*r* = 0.38). Thus, for example, hip and spine BMD had 6.8% associated SNPs in common, corresponding to ρ_P_ = 0.55 and ρ_G_ = 0.66 between them. Fewer SNPs were associated with both BMD and any of the hip geometric traits (eg, femoral neck and shaft width, section moduli, neck shaft angle, and neck length); ρ_G_ between BMD and geometric traits ranged from −0.24 to +0.40. In conclusion, we examined relationships between osteoporosis-related traits based on genome-wide associations. Most of the similarity between the quantitative bone phenotypes may be attributed to pleiotropic effects of genes. This knowledge may prove helpful in defining the best phenotypes to be used in genetic studies of osteoporosis. © 2010 American Society for Bone and Mineral Research.

## Introduction

Age-related osteoporotic fractures are common in the United States and represent a major public health threat that is likely to increase in importance as the population ages.(1) Bone mineral density (BMD), bone quantitative ultrasound (QUS), and bone geometry predict the risk of osteoporotic fractures(2–5); therefore, these traits are reliable proxies for fractures. Combinations of BMD assessments in more than one region,(6) as well as composite use of BMD with QUS(3) or BMD and hip geometry,(7) have been suggested to improve risk assessment in clinical practice. Studies over recent decades have documented the major contribution of genes to BMD,(8,9) QUS,(10) and bone geometry.(11,12) Osteoporotic fracture is also a heritable phenotype; however, there are indications that it is partly governed by genetic factors distinct from bone mineralization measured by BMD.(13–15) Moreover, genetics of the osteoporotic fracture phenotype is a difficult subject to study: Fractures typically do not occur until later in life. Therefore, quantitative risk factors (proxies) traditionally are used,(13,14,16) especially because these quantitative traits may be measured at any age. On the other hand, it is important to realize that none of these proxies is a perfect phenotype of osteoporosis, especially for a genetics study.

The entire body of knowledge about skeletal genetics has relied on the study of individually selected phenotypes. Thus candidate gene studies and previous genome-wide association study (GWAS) approaches typically are performed using one or a couple of skeletal phenotypes such as BMD of the spine and BMD of the hip. When significant findings are observed at one skeletal site but not the other,(17,18) this is often either minimized in importance or even held as evidence that genes operate differently at different skeletal sites, even though these sites have the same types of bone. Without a better approach in the future, there will continue to be uncertainty about such discordant findings. In terms of quantitative genetic theory, pleiotropy among the traits is expected to assist phenotype prioritization in the study (to decide which is the best phenotype for a planned genetic study); this is imperative for clearly distinguishing signal from noise in genetic association studies. Of note, prior linkage analyses revealed mostly skeletal site-specific quantitative trait loci (QTLs), suggesting that minimal genetic pleiotropy (shared genetic determinants) exists between cancellous and cortical BMD,(19) femoral and spinal BMD,(20,21) and BMD and calcaneal QUS.(22) These findings are rather unexpected given the apparent relatedness of these traits(23) and again raise the question of which one of the osteoporosis-related phenotypes is the most reliable for uncovering genetic effects.

GWASs using high-density genotyping platforms offer an unbiased strategy to identify new candidate genes for osteoporosis and also provide a unique opportunity to use phenotypic relations to discern the underlying biology (phenomic approach)(24–28); this can be applied to define the best phenotype in the pathway to fracture. In our GWASs for osteoporosis-related traits in the Framingham sample,(29) significant genetic associations with BMD phenotypes did not overlap with bone geometric phenotypes. The results of more recent GWASs of bone traits are similarly intriguing in that there is an incomplete overlap between association results for two of the most often studied skeletal sites: femoral neck and lumbar spine.(30,31) Since bone at different skeletal sites likely would be influenced by proteins in a more global fashion, this site specificity of genetic associations has been unanticipated.

Van Driel and colleagues(32) found that similarity between phenotypes was positively correlated with relatedness at the level of protein sequence and functional annotation. We similarly hypothesized that correlated phenotypes would share multiple associated single-nucleotide polymorphisms (SNPs). Evidently, there is also an effect of shared environmental factors.(33) The aims of this study were therefore to compare SNP associations for pairs of bone measurements with phenotypic correlations between them. We hypothesized that correlated bone phenotypes would share SNPs more frequently than by chance (and thus would be shown to be associated with pleiotropic susceptibility loci). We further decomposed the phenotypic correlations between the quantitative bone measures into genetic and environmental components to explore genetic and environmental sources of between-trait similarities.

We focused on women because osteoporotic fractures are more common in women and because genetic regulation has been shown to differ by gender.(34,35)

## Materials and Methods

### Sample

The sample used for our analyses was derived from two cohorts of the population-based Framingham Heart Study (FHS). Details and descriptions of the Framingham Osteoporosis Study (FOS) have been reported previously.(20,36) Description of the family samples with bone phenotypes available for the analyses in the FOS are provided elsewhere(37,38) and are available publicly through the Database of Genotype and Phenotype (dbGaP) at http://view.ncbi.nlm.nih.gov/dbgap. In brief, the original and the offspring cohorts in the FOS represent members of two-generational (mostly nuclear) families recruited at different times. The following analyses focused on the subsample of women. The study was approved by the Institutional Review Boards for Human Subjects Research of Boston University and Hebrew SeniorLife.

#### Osteoporosis-related skeletal phenotypes

In brief, the following measures were available in members of both cohorts of the FHS:

*Bone mineral density (BMD):* The participants underwent bone densitometry by dual-energy X-ray absorptiometry (DXA) with a Lunar DPX-L device (Lunar Corp., Madison, WI, USA) between 1996 and 2001. The coefficients of variation (*CV*%) in normal subjects for the DPX-L have been previously noted to be 0.9% for the lumbar spine (LS), 1.7% for the femoral neck (FN), and 2.5% for the trochanter.(36)

*Quantitative ultrasound (QUS):* QUS of the right heel was performed to obtain calcaneal broadband ultrasound attenuation (BUA) and speed of sound (SOS) with a Sahara bone sonometer (Hologic, Inc., Waltham, MA, USA) between 1996 and 2001. Based on duplicate same-day measurements on 29 subjects, *CV* values for BUA and SOS were 5.3% and 0.4%, respectively.(39)

*Hip geometry:* DXA scans were measured with an interactive computer program.(40) The program derived a number of proximal femoral structural variables, including gross anatomic femoral neck length (FNL) and neck shaft angle (NSA), as well as cross-sectional indices subperiosteal diameter (width, cm), cross-sectional bone area (CSA, cm^2^), and section modulus (*Z*, cm^3^), at the two femoral regions [narrow neck (NN) and the femoral shaft (S)]. *CV* values were reported previously to range from 3.3% (NN outer diameter) to 9.1% (FNL).(40)

*Other measurements (covariates)*: Information on age, sex, height, body mass index (BMI), and menopausal status and estrogen use were obtained for each woman at the time of the bone measurement. Women were assigned to one of two “estrogenic status” groups: (1) premenopausal or postmenopausal on estrogen (estrogen-replete) or (2) postmenopausal not on estrogen (estrogen-deplete). Details for these measurements are available elsewhere.(36,41)

#### Genotyping, Quality Control, and Population Substructure

Genotyping was conducted through the FHS SNP Health Association Resource (SHARe) project initiated in 2007 on all FHS participants with DNA available using the Affymetrix (Affymetrix Inc., Santa Clara, CA, USA) 500K (250K Sty and 250K Nsp) mapping array in addition to the Affymetrix 50K supplemental array (50K MIP). Sample-level exclusions included a participant call rate less than 97%, a per-subject heterozygosity that was ±5 SD from the mean, or a per-subject number of Mendelian errors greater than 165 (99th quantile). Genotyping from 433,510 SNPs in 8481 individuals passed these quality-control measures. Principal-components analysis was applied to evaluate population structure (infer axes of variation) using a subset of 425,173 SNPs with minor allele frequency (MAF) ≥ 0.01, Hardy-Weinberg equilibrium (HWE) *p* ≥ 10^−6^, and call rate ≥ 0.95. SNP weights for 10 principal components (PCs) were calculated using a maximal set of independent individuals (*n* = 882); the PCs for the remaining individuals were computed using the SNP weights obtained from this set of unrelated individuals. Since the first 4 of the 10 PCs were significantly associated with some bone traits (*p* < .01), we adjusted for PCs 1 through 4 in the SNP association analyses.

### Statistical analysis

Multivariable regression analysis was performed in women from each cohort (original and offspring) in order to obtain normalized or ranked residual phenotypes adjusted for covariates. Cohort-specific residuals were combined in ensuing analyses. Bone geometry traits were adjusted for age, age^2^, height, BMI, PCs 1 through 4 (the first four principal components), and estrogenic status. In the case of BMD and ultrasound variables, alcohol intake and smoking status were additionally adjusted.

*Bivariate variance component analyses (VCA):* VCAs were performed using the computer package Sequential Oligogenic Linkage Analysis Routines, Version 2.0 (SOLAR Southwest Foundation for Biomedical Research, San Antonio, TX, USA).(42) The correlation coefficient (ρ_P_) between any pair of traits is given by


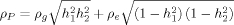
(1)

where ρ_G_ and ρ_E_ represent the shared additive genetic and environmental influences, respectively, whereas 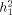 and 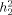 are the heritabilities of each trait. For the osteoporosis-related traits, ρ_E_ would include all nongenetic factors similar between relatives, such as effects of household, diet, exercise, and other environmental factors influencing bone density or skeletal dimensions. More extensive details regarding the development, implementation, and power of bi- and multivariate extensions to heritability analyses have been published elsewhere.(43,44)

The sample for bivariate VCA included up to 1473 genotyped FHS women from 323 to 327 extended families, with family sizes ranging from 2 to 29 individuals, who had BMD, QUS, and geometry measurements.

*Genome-wide association study (GWAS):* We performed GWAS analyses using population-based additive linear mixed-effects (LME) models(45) with 433,510 SNPs in the sample of 2073 women. LME regression models adjust for correlations owing to family relationships in pedigrees of arbitrary sizes and varying degrees of relationship.

*GWAS database mining:* Finally, we searched for SNPs nominally associated (*p* < .01) with more than one trait from the list of bone-related phenotypes in LME analyses. We evaluated all possible pairs of bone phenotypes by comparing ρ_P_, ρ_G_, and ρ_E_ among each pair of traits (absolute values) with the proportion of SNPs associated with both these traits at the preceding alpha level. To assess the overlap between the phenotypes in a more quantitative way, we developed a simple measure of genetic similarity between members of the pair of phenotypes, a percentage of shared association in LME analysis, calculated as the number of shared associated SNPs divided by the total number of non-union-associated SNPs times 100 at α threshold 0.01. Under assumption that the traits are independent, this *p* value for a single-trait-association corresponds to its square, *p* = .0001, for the pair of traits.

We conducted simulations in order to evaluate observed amount of shared associations against expected values under the null hypothesis of no association. Thus associations between simulated SNPs and each pair of bone phenotypes were performed to obtain a null distribution (20,000 repetitions). If the number of observed SNPs associated (at a specified α value of 0.01) with a pair of traits was larger than a threshold set by simulation tests (the number of associations expected by chance), we considered this evidence of an SNP having a pleiotropic effect on both phenotypes.

## Results

Genome-wide association analysis by LME models resulted in SNPs associated with the studied traits at the lowest *p* values ranging from *p* = 1.45 × 10^−5^ to *p* = 4.15 × 10^−8^ (Table 1). At a less strict threshold of statistical significance, α = 0.01, there were from 4189 SNPs for NSA to 5012 SNPs associated with FN BMD (Table 1).

**Table 1:** Number of Significant Associations for the Individual Phenotypes (LME Results)

Trait	α = 0.01	Lowest *p* value
FN BMD	5012	2.56 × 10^−6^
LS BMD	4806	1.45 × 10^−5^
BUA	4437	5.25 × 10^−6^
SOS	4779	3.43 × 10^−7^
NSA	4189	7.98 × 10^−7^
FNL	4372	5.57 × 10^−7^
NN width	4281	4.15 × 10^−8^
Shaft width	4272	8.78 × 10^−6^
Shaft section modulus	4239	1.87 × 10^−7^

Based on the null distribution from the simulation analyses (20,000 repetitions), we found slightly more associated SNPs to be shared between traits under the null hypothesis than would be expected (Table 2). The average ratio of observed to predicted bivariate associations was 1.18, with a minimum of 0.68 (LS BMD and shaft width: expected 55, observed 37) and a maximum of 1.94 (BUA and NN width: expected 38, observed 75).

**Table 2:** Number of Expected and Observed Significant Associations Shared Between the Phenotypes at α = 0.01

	FN BMD	LS BMD	BUA	SOS	NSA	FNL	NN width	Shaft width	Shaft section modulus
FN BMD	5012	489	211	210	35	44	76	48	197
LS BMD	622	4806	202	205	43	45	47	55	162
BUA	263	245	4437	1791	44	43	38	48	70
SOS	353	293	2017	4779	44	46	43	49	57
NSA	41	51	53	37	4189	36	47	49	45
FNL	43	34	62	57	40	4372	150	200	138
NN width	90	53	75	60	50	194	4281	515	126
Shaft width	90	38	66	60	37	207	529	4272	319
Shaft section modulus	212	144	64	84	47	138	106	347	4239

*Note:* Above diagonal = expected number of shared significant associations at α = 0.01; below diagonal = observed number of shared significant associations; diagonal = number of significant associations for the individual phenotypes.

**Fig. 1:**
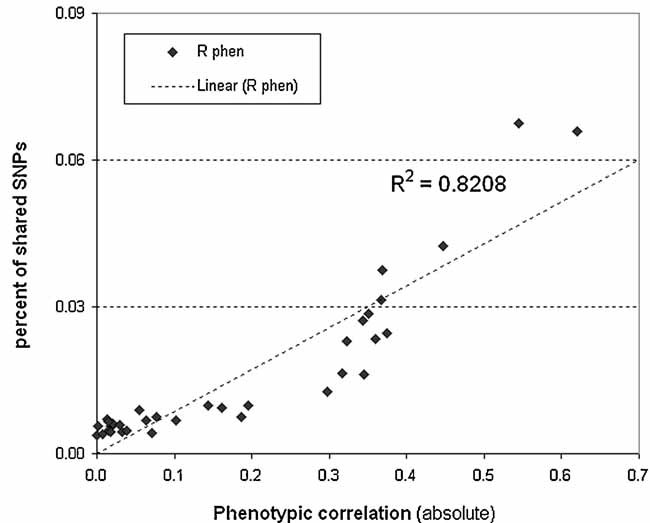
Relationship between the phenotypic correlation (ρ_P_) and observed proportion of SNPs shared between traits at α = 0.01.

Figure 1 illustrates the relationship between the phenotypic correlations (ρ_P_) and observed proportions of SNPs shared between traits at α = 0.01. Correlation between ρ_P_ and the proportion of the associated SNPs was *r* = 0.91. Pairs of BMD and QUS traits were characterized by phenotypic correlations ranging from 0.85 (BUA and SOS) to 0.34 (BUA and LS BMD). Similarly, correlations among the hip geometry traits ranged from *r* = 0.62 between NN width and shaft width to 0.29 between shaft width and shaft *Z*. Correlations between bone mass and cross-sectional hip geometry indices ranged from +0.36 between LS BMD and shaft *Z* to −0.20 between FN BMD and NN width. Notably, indices of femoral shape, NSA, and neck length were neither significantly correlated among themselves nor with other traits. Not surprisingly, the number of associated SNPs was highest for the combination BUA and SOS (2017 SNPs) given the exceptionally high correlation between these measures and because both indices are measured on the same bone. We excluded this pair from the following analyses. Highly correlated LS and FN BMD had 622 associated SNPs in common (6.7% at *p* < .01); similarly, NN width and shaft width shared 529 SNPs (6.6%). Fewer SNPs were associated with both BMD and hip geometric traits (femoral neck–shaft angle, neck length, neck and shaft width, section modulus). Also, bone geometry traits shared a relatively small percentage of associated SNPs among themselves (except from 6.6% between NN width and shaft width).

We then asked whether the coincidence of associations could be predicted by the shared genetic (ρ_G_) or environmental correlations (ρ_E_). Similar to the phenotypic correlations, ρ_G_ between BMD and QUS traits ranged from 0.43 (BUA and LS BMD) to 0.66 (NN BMD and LS BMD) (Table 3). We excluded the pair of SOS and BUA from consideration owing to an extremely high ρ_G_ (0.98). Genetic correlations were of similar magnitude among hip geometry traits (ρ_G_ ranging from 0.53 to 0.91) but lowest for NSA with other femoral geometric traits (ρ_G_ ranging from 0.06 to 0.22). Genetic correlations generally were lower between hip geometry and BMD or QUS traits (highest ρ_G_ = 0.40 between LS BMD and shaft *Z*). Environmental correlations ranged from +0.74 (SOS and BUA) to −1.0 (shaft width and NN width).

**Table 3:** Matrix of the Genetic and Environmental Correlations Between Bone Traits

	FN BMD	LS BMD	BUA	SOS	NSA	FNL	NN width	Shaft width	Shaft section modulus
FN BMD	0.455	0.426	0.280	0.297	−0.080	−0.208	−0.346	0.113	0.499
LS BMD	0.659	0.634	0.258	0.169	−0.064	−0.195	−0.622	0.327	0.264
BUA	0.435	0.425	0.483	0.744	0.052	−0.254	−0.458	−0.325	0.189
SOS	0.457	0.551	0.975	0.451	−0.102	−0.237	−0.489	−0.437	0.061
NSA	0.046	0.031	−0.037	0.183	0.306	−0.167	−0.446	−0.270	−0.199
FNL	0.240	0.112	0.164	0.159	0.222	0.649	−0.526	−0.790	−0.145
NN width	−0.193	0.086	0.027	0.037	0.110	0.528	0.947	−1.000	−0.996
Shaft width	−0.241	−0.151	−0.037	−0.035	0.058	0.660	0.906	0.948	−0.443
Shaft section modulus	0.276	0.395	0.183	0.242	0.214	0.575	0.570	0.673	0.677

*Note:* Above diagonal = environmental correlations (ρ_E_); below diagonal = genetic correlations (ρ_G_); diagonal = heritabilities from univariate polygenic analysis (all significant at *p* < .0001).

**Fig. 2:**
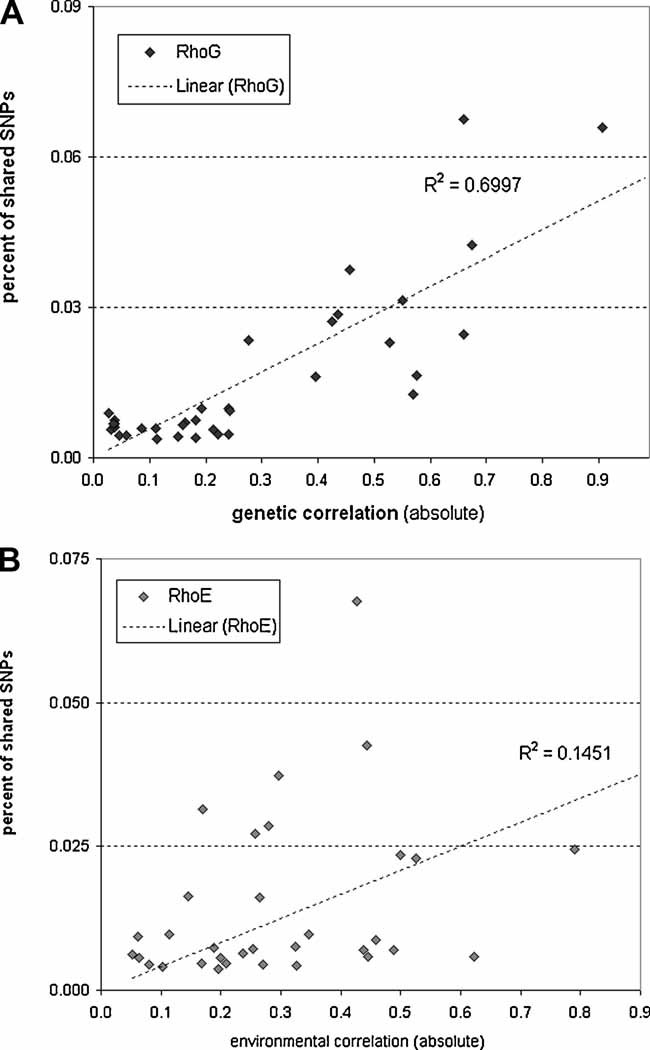
Relationship between the genetic (ρ_G_) or environmental correlation (ρ_E_) and observed proportion of SNPs shared between traits at α = 0.01

Figure 2 illustrates the relationship between the genetic and environmental components of the phenotypic correlations and observed proportions of shared SNPs at α = 0.01. Correlation between the ρ_G_ and the proportion of SNPs was substantial (*r* = 0.84; Fig. 2*A*). Notably, correlation between the ρ_E_ and the proportion of the SNPs was much lower (*r* = 0.38; Fig. 2*B*).

## Discussion

In this study we attempted to evaluate how much of the similarity between quantitative bone phenotypes may be attributed to pleiotropic effects of genetic variants genome-wide. Our results indicate that phenotypic and genetic correlations between a pair of bone phenotypes correspond to the proportion of associated polymorphisms shared by the two traits (*r*^2^ = 0.82 and 0.70, respectively). Environmental correlation is also related to the proportion of the SNPs, but its relationship with the number of shared SNPs was weaker (*r*^2^ = 0.15). Based on the results of simulation tests, we observed a slightly higher proportion of SNPs than would be expected to be shared between traits under the null hypothesis (ratio of observed to expected = 1.18). Therefore, at least 18% of SNPs identified by our GWASs may be thought of as being truly pleiotropic. The importance of these findings is that multiple traits should be studied to better understand the site specificity of the genetic determinants of bone phenotypes. On the other hand, the challenge for the field is to be able to capture the moderate percentage of variation in different skeletal phenotypes that is under the regulation of the same genes. Contrasting pleiotropic with nonpleiotropic genes for skeletal phenotypes inevitably will yield new molecular insights into basic bone biology.

An interesting finding of this study was that the genetic correlations *within* the BMD phenotypic group were greater than those *between* BMD and heel ultrasound and femoral geometric traits, suggesting that different sets of genes contribute to BMD of the hip and spine compared with that of the heel and hip geometry. Moreover, high genetic correlations between LS and FN BMD (ρ_G_ = 0.66) corresponded to a relatively high proportion of genome-wide SNPs that were significantly associated with both measures (6.7%). Indeed, these genetic results match the emerging epidemiologic knowledge: Thus several recent studies concluded that a combination of BMD measurements at the lumbar spine and femoral neck predicts *any* osteoporotic fracture independent of the fracture's skeletal location.(4,5) BMD measurements at the lumbar spine predict osteoporotic fracture as good as measurements at the femoral neck, suggesting that these measures are indeed interchangeable as risk factors for the fracture.(5) Since relations between phenotypes often reflect a biologic and functional interaction at the gene level, we believe that LS and FN BMD are indeed genetically alike. Along these lines, since BMD and hip geometry do not share many genetic associations (and thus are genetically different), there is an incentive to combine them for fracture prediction (as suggested by refs. (7) and (46)) given that each trait reflects a unique aspect of bone biology.

Some of the relationships between the skeletal traits studied are similarly worthy of mention. Given the high collinearity between measures of bone ultrasound, BUA and SOS, it was not surprising to observe that they shared the highest number of associated SNPs (28%). High genetic correlation between NN width and shaft width (ρ_G_ = 0.91) is expected, given that both measures of bone width are a function of allometric growth early in ontogenesis and a periosteal apposition later on. Even a substantial ρ_G_ between neck length and both NN width and shaft width (ρ_G_ = 0.53 and 0.66, respectively) is not surprising given the cantilever functioning of the femoral neck during locomotion. Wide bone, less prone to bending, needs to counterweight the long neck, a known risk factor for fracture.(47) This might be triggered by a perturbation in circumscribed genetic pathway. For example, delayed mineralization of femoral growth plate cartilage(48,49) may lead to elongation of the femoral neck (with reduction of the neck shaft angle and height of the greater trochanter); both changes prompt functional adaptations of muscles to maintain fitness in walking and running,(50) which, in turn, triggers ossification of entheses and periosteal outgrowth. The latter will appear as larger width of the bone (reviewed in ref. (51)).

In order to determine the nature of the shared SNPs, we extracted a list of SNPs associated at *p* < .001 with both FN and LS BMD. From Table 4 it follows that there are 38 SNPs (some SNPs in linkage disequilibrium with *r*^2^ ≥ 0.5) found on 14 chromosomes. These SNPs are in or near the following genes: *RAPGEF4*, *ANKRD17*, *SLC35F1*, *PTPRD*, *ITIH5*, *LMO1*, *NELL1*, *TTC23*, and *CD300E*, as well as chromosome 2 *Orf34*. Other SNPs were found in the vicinity of *TMEM70*, *CADM4*, *COX18*, *FAM13C1*, *IRAK2*, *CBLN1,* and other genes. Notably, β coefficients are consistent in their sign and similar for both BMD traits (which suggests that these SNPs indeed may be pleiotropic). Whether the selected genes are truly associated with osteoporosis can only be shown functionally, and it is not possible to validate our approach to selection of presumably pleiotropic candidate genes without further empirical research.

**Table 4:** Polymorphisms Significantly (*p* < .001) Associated With Both Lumbar Spine and the Femoral Neck BMD

					Femoral neck			Lumbar spine		
						
SNP	Chr.	Position^a^	In gene	Neighboring genes (60 kb from SNP)	Beta^b^	SE beta	*p* Value	beta	SE beta	*p* Value
rs6733708	2	37814216		*CDC42EP3*	−0.135	0.034	6.28E-05	−0.112	0.034	9.71E-04
rs698798*	2	44544018	*C2orf34*		−0.213	0.057	2.02E-04	−0.194	0.058	8.19E-04
rs698819*	2	44566433	*C2orf34*		−0.214	0.059	3.05E-04	−0.258	0.060	**1.67E-05**
rs3769292	2	173434505	*RAPGEF4*		−0.197	0.053	1.88E-04	−0.176	0.053	9.01E-04
rs1569159	2	181189870		*UBE2E3*	0.183	0.056	9.91E-04	0.192	0.056	5.69E-04
rs16864755	2	223982866		*SCG2*	0.241	0.052	**3.86E-06**	0.187	0.053	4.23E-04
rs457414	3	10177884		*C3orf24;VHL;FANCD2;IRAK2;C3orf10*	−0.114	0.034	6.69E-04	−0.113	0.034	8.61E-04
rs9846680	3	179603561		*KCNMB2*	−0.230	0.060	1.20E-04	−0.238	0.060	7.60E-05
rs1545026	3	196061661		*FAM43A*	0.169	0.048	4.97E-04	0.180	0.049	2.15E-04
rs13148678*	4	74077760		*COX18*	0.161	0.044	2.40E-04	0.166	0.044	1.85E-04
rs7664273*	4	74226102	*ANKRD17*		0.149	0.043	5.71E-04	0.149	0.044	6.59E-04
rs13119179*	4	74262694	*ANKRD17*		0.144	0.043	8.73E-04	0.149	0.044	6.48E-04
rs283062	6	118728149	*SLC35F1*		0.119	0.033	2.89E-04	0.115	0.033	5.00E-04
rs665506	7	132023073		*PLXNA4*	0.126	0.034	2.17E-04	0.126	0.034	2.36E-04
rs1905045	8	75062568		*TCEB1;LY96;TMEM70*	0.162	0.038	**2.03E-05**	0.164	0.038	**1.91E-05**
rs2317356*	8	137106399		*KHDRBS3*	0.131	0.035	2.06E-04	0.119	0.036	8.16E-04
rs2317355*	8	137106698		*KHDRBS3*	0.137	0.035	1.03E-04	0.121	0.036	7.03E-04
rs1031282*	8	137123839		*KHDRBS3*	0.131	0.035	2.17E-04	0.121	0.036	7.52E-04
rs1332199*	9	9493477	*PTPRD*		0.154	0.042	2.44E-04	0.146	0.042	5.59E-04
rs639168*	9	9529574	*PTPRD*		0.178	0.047	1.74E-04	0.193	0.048	**4.93E-05**
rs668026*	9	9571692	*PTPRD*		0.122	0.034	3.78E-04	0.120	0.035	5.21E-04
rs681437*	9	9572128	*PTPRD*		0.124	0.034	3.05E-04	0.123	0.035	3.92E-04
rs598768*	9	9579682	*PTPRD*		0.127	0.033	1.10E-04	0.110	0.033	9.41E-04
rs657849*	9	9583722	*PTPRD*		0.140	0.033	**2.39E-05**	0.112	0.033	7.70E-04
rs1889524*	10	7657473	*ITIH5*		−0.107	0.032	8.43E-04	−0.123	0.032	1.57E-04
rs1537631*	10	7657765	*ITIH5*		−0.119	0.032	2.47E-04	−0.129	0.033	9.62E-05
rs2275069*	10	7658692	*ITIH5*		−0.117	0.033	3.74E-04	−0.137	0.033	**3.82E-05**
rs384626	10	60806221		*FAM13C1*	−0.110	0.031	4.36E-04	−0.105	0.032	9.32E-04
rs7911563	10	60878297		*FAM13C1*	−0.118	0.032	2.52E-04	−0.110	0.033	7.19E-04
rs453061	11	8227452	*LMO1*	*LMO1*	0.490	0.142	5.47E-04	0.470	0.142	9.56E-04
rs10766761	11	20972609	*NELL1*	*NELL1*	−0.120	0.036	7.90E-04	−0.130	0.036	3.30E-04
rs1968699	15	97529279	*TTC23*	*TTC23;DMN*	−0.240	0.068	4.46E-04	−0.254	0.070	2.65E-04
rs2216263	16	47911005		*CBLN1;C16orf78*	0.109	0.033	9.24E-04	0.127	0.033	1.28E-04
rs1389529	16	53286410		*IRX5*	0.224	0.066	7.16E-04	0.259	0.066	9.59E-05
rs1699607	17	70130820	*CD300E*	*RAB37;C17orf77*	−0.108	0.032	6.73E-04	−0.106	0.032	9.18E-04
rs892583	18	43170372		*IER3IP1*	0.134	0.036	2.29E-04	0.121	0.036	9.11E-04
rs740586	19	48888371		*CADM4;IRGC;C19orf61;PLAUR*	0.126	0.038	8.71E-04	0.128	0.038	7.70E-04
rs346062	19	48890417		*CADM4;IRGC;C19orf61;PLAUR*	−0.131	0.032	**3.53E-05**	−0.107	0.032	7.53E-04

a NCBI build *35* position.

b βcoefficient in the additive model.

* Neighboring SNPs in linkage disequilibrium (*r*^2^ ≥ 0.5).

One important observation of this study was that genetic correlations (ρ_G_) obtained by bivariate variance components analysis for a pair of traits seemed to reliably predict the percentage of SNPs associated with both traits at a magnitude similar to the phenotypic correlation among them. The correlation between the ρ_G_ and proportion of SNPs associated with a pair of traits was substantial (*r* = 0.84). Still, the genetic correlations did not perfectly match the observed pleiotropic associations. There are differences in the analytic approaches that may explain a nonperfect correlation between the two metrics of pleiotropy. First of all, genetic correlations are calculated based on similarity among family members, with the assumption of multiple additive alleles, whereas genome-wide association treats each allele as independent of others. ρ_G_ may be biased upward because it does not take into account nonadditivity of genetic effects (the possibility of imprinting, epigenetic and dominance effects, as well as interactions among genes that contribute to functional integration of morphologic traits(52)).

Recent GWASs of nonbone phenotypes similarly noted that a substantial proportion of trait heritability remains to be characterized; this phenomenon, so-called genetic dark matter, is reviewed in ref. (53). There is good reason to assume that this “dark matter” is neither an illusion created by inflated estimates of heritability nor the consequence of marked nonadditivity of effects, but a function of genome coverage by only common SNPs. Indeed, we analyzed only diallelic SNPs with the minor allele frequency of 1% or more and no other type of genetic variance. Furthermore, despite good coverage of the genome in general, a known limitation of the Affymetrix 500K SNP GeneChip is its sparse coverage of coding regions, which may result in losing some important associations. This limitation was somewhat overcome by the addition of approximately 50,000 gene-centric SNPs; however, there are still regions with imperfect coverage.

This study has several limitations. It is recognized that the genetic contribution to fracture is not fully explained by bone strength factors per se; other important risk factors, such as measures of muscle strength, neurosensory functioning, and balance, need to be added to the preceding skeletal phenotypes in order to characterize fracture in its entirety. A phenomic approach(54,55) is expected to help overcome current controversies by comparing the genetic findings of the preceding combination of traits with those of the fracture. Our future study therefore will attempt to solve the following question: Do body size components, such as height, amount of lean mass, and long bone lengths, have different or similar genetic architecture with the bone mass traits?(56) And we will approach this by looking into the relationship between body composition traits and osteoporosis phenotypes.

Furthermore, an estimate of the overall degree of pleiotropy in osteoporosis-related traits generally was higher than can be explained by random chance based on our simulations. However, calculation of the numbers of statistically significant results expected by chance is complicated by the interrelatedness of the variables,(57) as well as by linkage disequilibrium between the SNPs.(58) At present, we are performing simulations with various thresholds of linkage disequilibrium. Finally, the relationship between genetic correlation and the observed proportion of significantly shared SNPs did not take into account whether or not the effect of the SNP was in the same or in the opposite direction; at present, we restricted the analysis by the absolute values of ρ_P_, ρ_G_, and ρ_E_ and ignored the direction of SNP effect.

Despite of these limitations, one of the unique aspects of the FOS is that we can apply both family-based variance decomposition methods and population-based GWAS techniques. Compared with our previous GWASs,(29) the current sample is close to three times the size of that one, and the genotyping is fivefold denser. Moreover, in future studies, we can extend this approach to newer musculoskeletal imaging modalities, as well as to longitudinal phenotypes.

The implications of this study are twofold: First, we outlined phenotypic and genetic relationships between the multiple osteoporosis-related quantitative phenotypes. Second, we found a set of putative pleiotropic polymorphisms for these traits. Results of our study are especially important in lieu of the complexity of the quantitative phenotype of fracture for genetic studies of osteoporosis. Given the current controversy about whether the same genes that are associated with the proxy phenotypes, such as BMD or bone geometry, are also related to osteoporotic fracture, GWAS results may be used to better understand the degree to which bone phenotypes share genetic determinants among themselves as well as with osteoporotic fractures, a question still unanswered.(13,14)

This is the first phenomic scan that used GWASs of multiple osteoporosis-related phenotypes to discover pleiotropic genetic variants. These results can be used for data reduction and for choosing best phenotype(s) for genetic research in osteoporosis, as well as to inform the ever-growing field of genetic epidemiology, with GWASs of multiple related phenotypes available in the same data set. With the availability of newer imaging modalities, the number of refined osteoporosis-related phenotypes will increase, and debates will intensify as to what proxies for osteoporosis are the most promising for genetic research.

In summary, knowledge of the genetic predisposition to low bone mass or less favorable geometry is important for diagnostic purposes, personalized medicine, and treatment monitoring because these measures are quantitative traits reliably measured at any age rather than dichotomous later-life events such as fracture. A greater integration of medicine and biology calls for innovative computational and informatics tools and high-throughput discovery technologies,(59) especially for complex heritable diseases such as osteoporosis. We showed that correlated bone phenotypes share SNPs more frequently than by chance, therefore suggesting that pleiotropic genetic factors may govern susceptibility to fracture. Since mapping genetically correlated phenotypes should result in increased power,(60,61) analyzing a subset of highly correlated traits should be a successful strategy to further explore genetic “dark matter.” Future GWASs should be designed in such a way as to have the best chance of locating these pleiotropic genes.
